# Visions of Matchstick Men and Icons of Industrialization

**DOI:** 10.3201/eid2311.AC2311

**Published:** 2017-11

**Authors:** Byron Breedlove

**Affiliations:** Centers for Disease Control and Prevention, Atlanta, Georgia, USA

**Keywords:** art science connection, emerging infectious diseases, art and medicine, about the cover, Visions of Matchstick Men and Icons of Industrialization, Going to Work, Laurence S. Lowry, *Legionella*, bacteria, Legionnaires’ disease, legionellosis, pneumonia

**Figure:**
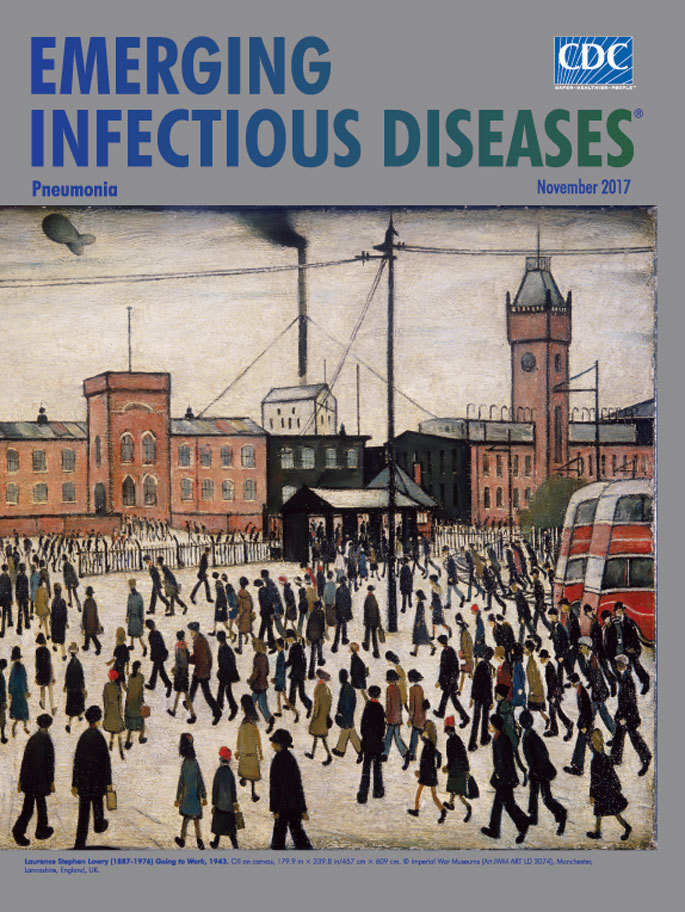
**Laurence Stephen Lowry (1887–1976) Going to Work, 1943.** Oil on canvas, 179.9 in × 239.8 in/457 cm × 609 cm. AM1976–861. Imperial War Museums, © IWM (Art.IWM ART LD 3074), Manchester, Lancashire, England, UK.

English artist Laurence Stephen Lowry, more commonly referred to as L.S. Lowry, is remembered for being enigmatic and mischievous. Lowry, who rejected 5 different honors during his lifetime—including an Officer of the Most Excellent Order of the British Empire in 1955 and knighthood in 1968—holds the record for the most rejected British honors.

Born in 1887, Lowry lived an upscale area of south Manchester, UK, until financial difficulties forced his parents to relocate to an industrial part of Salford, which he initially hated. His feelings slowly changed: “After a year I got used to it. Within a few years I began to be interested and at length I became obsessed by it.”

Though Lowry struggled with schoolwork, he enjoyed drawing and as a youth spent his own money on private art lessons. In 1905, he started taking evening classes in Manchester, where he studied with the French artist Adolphe Valette, who introduced him to Impressionism. Lowry continued attending evening classes throughout the next two decades.

Despite his enthusiasm for art, Lowry worked as a rent collector for 42 years, retiring from the Pall Mall Property Company on his 65th birthday. His day job accorded Lowry up-close access to everyday life. Jackie Wullschlager, chief art critic of the *Financial Times,* observed that Lowry “knew quotidian dreariness at close hand; he was also used to keeping accounts, and his paintings display an unsentimental, ledger-like notation of pictorial facts even as they become more compositionally complex.”

Lowry’s best known works depict the environs and icons of industrialization such as mills, manufacturing plants, bridges, and railway stations. Smokestacks and chimneys atop factories and houses belch black smoke into the hazy white sky Lowry favored. His teeming human figures—commonly called “matchstick men” for their singular narrow vertical forms—rigidly move about their activities, appearing disconnected from each other despite their proximity in crowds and long lines.

Focused, perhaps obsessed, with maintaining precision in color and tone, Lowry used only Winsor & Newton Winton Oil Color paints. Further, he famously adhered to a five-color palette of the company’s paints, consisting of Ivory Black, Vermillion, Prussian Blue, Yellow Ochre, and Flake White.

*Going to Work*, this month’s cover art, was created to fulfill a short-term commission from the War Artists Advisory Committee. It falls precisely within Lowry’s principal oeuvre. The artist fills the foreground with countless anonymous factory workers trudging through the snow, dutifully coalescing into distinct queues as they march toward the bulwark of grim buildings to begin manufacturing machinery and motors at the iconic Mather & Platt foundry, Manchester (which continued operating in some capacity under various owners until July 2017). Crowded between and before the buildings, numerous less-distinct figures swell the ranks of workers toiling to support the war effort.

Viewers, as are Lowry’s workers, are inevitably drawn toward the row of buildings dominating the center of the painting. A single smudge of green partly obscured by the double-decker bus suggests a lone tree. Wires stretch across the top of the canvas, and hovering ominously overhead, a pair of barrage balloons offers modest defense from aerial attacks on the factory compound.

Lowry did not consider art a medium for agitation or activism. In his words, “To say the truth, I was not thinking very much about the people. I did not care for them the way a reformer does. They were part of a private beauty that haunted me. I loved them and the houses in the same way, as part of a vision. Had I drawn them as they are, it would not have looked like a vision.”

When Lowry died of pneumonia on February 23, 1976, he was both wealthy and well known as an artist and art collector. A few months after his death, the Royal Academy’s retrospective exhibition of his works achieved the record number of visitors for any exhibition by a British artist up to that time.

About 5 months after Lowry’s death, an outbreak of a new type of pneumonia, now known as Legionnaires’ disease, occurred among attendees at an American Legion convention in Philadelphia, Pennsylvania, USA. *Legionella* bacteria can cause Legionnaires’ disease or Pontiac fever, collectively known as legionellosis. *Legionella *bacteria are found naturally in freshwater environments but can infect humans when they grow and spread in manufactured water systems.

Viruses, bacteria, and fungi can all cause pneumonia. Many of those factory workers who inspired Lowry’s nameless matchstick men may have also died of the disease that ultimately took Lowry. In the 3rd edition of his classic textbook *The Principles and Practice of Medicine* (1898), Sir William Osler describes pneumonia as “the old man's friend,” because death from pneumonia seemed to involve less obvious agony than other common causes of death. Despite the availability of vaccines and antibiotics and the remarkable advances in respiratory care, pneumonia continues to affect hundreds of millions of people, old and young, in all parts of the world and also remains the single largest cause of child deaths worldwide.
